# A scoring system developed from a nomogram to differentiate active pulmonary tuberculosis from inactive pulmonary tuberculosis

**DOI:** 10.3389/fcimb.2022.947954

**Published:** 2022-09-02

**Authors:** Qi Yu, Jisong Yan, Shan Tian, Wujin Weng, Hong Luo, Gang Wei, Gangyu Long, Jun Ma, Fengyun Gong, Xiaorong Wang

**Affiliations:** ^1^ Department of Infectious Diseases, Wuhan Jinyintan Hospital, Tongji Medical College of Huazhong University of Science and Technology, Hubei Clinical Research Center for Infectious Diseases, Wuhan Research Center for Communicable Disease Diagnosis and Treatment, Chinese Academy of Medical Sciences, Joint Laboratory of Infectious Diseases and Health, Wuhan Institute of Virology and Wuhan Jinyintan Hospital, Chinese Academy of Sciences, Wuhan, China; ^2^ Department of Respiratory and Critical Care Medicine, Wuhan Jinyintan Hospital, Tongji Medical College of Huazhong University of Science and Technology, Hubei Clinical Research Center for Infectious Diseases, Wuhan Research Center for Communicable Disease Diagnosis and Treatment, Chinese Academy of Medical Sciences, Joint Laboratory of Infectious Diseases and Health, Wuhan Institute of Virology and Wuhan Jinyintan Hospital, Chinese Academy of Sciences, Wuhan, China; ^3^ Department of Infectious Diseases, Union Hospital, Tongji Medical College, Huazhong University of Science and Technology, Wuhan, China; ^4^ Department of Oncology, Quzhou Hospital of traditional Chinese Medicine, Zhejiang University of Chinese Medicine, Quzhou, China; ^5^ Department of Science and Education, Wuhan Jinyintan Hospital, Tongji Medical College, Huazhong University of Science and Technology, Wuhan, China; ^6^ Department of Laboratory Medicine, Wuhan Jinyintan Hospital, Tongji Medical College, Huazhong University of Science and Technology, Wuhan, China; ^7^ Department of Respiratory and Critical Care Medicine, Union Hospital, Tongji Medical College, Huazhong University of Science and Technology, Wuhan, China

**Keywords:** Active pulmonary tuberculosis, inactive pulmonary tuberculosis, nomogram, differential diagnosis, scoring system

## Abstract

**Purpose:**

This study aimed to develop and validate a scoring system based on a nomogram of common clinical metrics to discriminate between active pulmonary tuberculosis (APTB) and inactive pulmonary tuberculosis (IPTB).

**Patients and methods:**

A total of 1096 patients with pulmonary tuberculosis (PTB) admitted to Wuhan Jinyintan Hospital between January 2017 and December 2019 were included in this study. Of these patients with PTB, 744 were included in the training cohort (70%; 458 patients with APTB, and 286 patients with IPTB), and 352 were included in the validation cohort (30%; 220 patients with APTB, and 132 patients with IPTB). Data from 744 patients from the training cohort were used to establish the diagnostic model. Routine blood examination indices and biochemical indicators were collected to construct a diagnostic model using the nomogram, which was then transformed into a scoring system. Furthermore, data from 352 patients from the validation cohort were used to validate the scoring system.

**Results:**

Six variables were selected to construct the prediction model. In the scoring system, the mean corpuscular volume, erythrocyte sedimentation rate, albumin level, adenosine deaminase level, monocyte-to-high-density lipoprotein ratio, and high-sensitivity C-reactive protein-to-lymphocyte ratio were 6, 4, 7, 5, 5, and 10, respectively. When the cut-off value was 15.5, the scoring system for recognizing APTB and IPTB exhibited excellent diagnostic performance. The area under the curve, specificity, and sensitivity of the training cohort were 0.919, 84.06%, and 86.36%, respectively, whereas those of the validation cohort were 0.900, 82.73, and 86.36%, respectively.

**Conclusion:**

This study successfully constructed a scoring system for distinguishing APTB from IPTB that performed well.

## Introduction

Before the coronavirus disease pandemic, tuberculosis (TB) was one of the leading causes of mortality due to a single infectious agent. In 2020, there were approximately 10 million cases, nearly 5.8 million new cases of TB and 1.3 million fatalities from TB globally (World Health Organization, 2021), and more than 80% of these were pulmonary TB (PTB) (Cui et al., 2020). In China, according to the Expert Consensus on Diagnosis and Prevention of Inactive Pulmonary Tuberculosis published in China 2021 (Cheng et al., 2021) and the Expert consensus on a standard of activity judgment of pulmonary tuberculosis and its clinical implementation published in China 2020 (Deng and Lu, 2020), the PTB could be divided into active PTB (APTB) and inactive PTB (IPTB). APTB was defined as positive microbiological examination with/without typical symptoms, or clinical diagnosis (Deng and Lu, 2020). And the presence of abnormal stable radiography results in a person with a positive tuberculin skin test (TST) or interferon-release assay (IGRA), negative bacteriologic assay (if performed), and no clinical and/or radiographic evidence of current disease (after excluding other infections) is defined as inactive PTB (IPTB) (Goldberg et al., 1999). The classification is different from the classification provided by Drain PK in 2018, tuberculosis is divided into five categories, named eliminated TB infection, latent TB infection, incipient TB infection, subclinical TB disease and active TB disease, respectively (Drain et al., 2018). For reasons of different treatment regimens and duration, the spectrum of TB disease was divided into APTB and IPTB according to the presence or absence of radiological or microbiological evidence of M. Tuberculosis (Deng and Lu, 2020; Cheng et al., 2021). However, compared with patients with LTBI or incipient TB infection with no pulmonary abnormalities, patients with IPTB have a higher risk of developing APTB (Walter et al., 2014; Gao et al., 2018). China is a country with a high burden of TB. About a quarter of the population is infected with M. tuberculosis, but most of them do not progress to active TB in their lifetime. Therefore, providing TB preventive treatment for IPTB is more cost-effective and targeted, especially in remote areas and economically underdeveloped areas. Additionally, in clinical practice, for IPTB patients, abnormal pulmonary imaging has become the main reason for them to come to the hospital for help. Based on these, the differential diagnosis between APTB and IPTB is crucial for clinical decision-making, and once IPTB is identified, treatment strategies will change significantly. Delayed therapy in patients with APTB due to misdiagnosis results in disease progression, whereas overtreatment in patients with IPTB increases the cost burden, risk of adverse effects, and risk of drug resistance (Goldberg et al., 1999). Hence, it is critical to develop a promising method that can precisely discriminate between APTB and IPTB. Unfortunately, existing studies have focused on discriminating between active TB infection (ATBI) and LTBI, and no studies have aimed to distinguish between APTB and IPTB. Hence, there are still great limitations to distinguishing APTB from IPTB.

Five methods, namely serum biomarker examination, immunological examination, histopathological examination, laboratory microbiological examination, and imaging examination, are valuable for diagnosing APTB. However, these methods have certain limitations. Regarding serum biomarker examination, no single biomarker is effective in determining TB activity; hence, serum biomarker examination produces a low level of evidence in activity judgments. The tuberculin skin test (TST) and interferon-release assay (IGRA) are two major immunological examinations employed and included in the World Health Organization guidelines for the diagnosis of LTBI. However, neither test can distinguish between LTBI and ATBI (Wallis et al., 2013; Lewinsohn et al., 2017; Cohen et al., 2019), let alone APTB and IPTB. Histopathological and laboratory microbiological examinations are the gold standards for diagnosing APTB. However, histopathological examinations, such as invasive biopsies accompanied by trauma, are inconvenient for patients and in clinical practice. In addition, the sensitivity of laboratory microbiological examinations, including smear microscopy (Davies and Pai, 2008), Mycobacterium tuberculosis (MTB) culture (Seifert et al., 2021), and molecular detection by polymerase chain reaction (Noordhoek et al., 1994) or Xpert MTB/RIF (Trébucq et al., 2011; Ponnudurai et al., 2018), is unsatisfactory or limited. Karen et al. demonstrated that 50% of patients had negative results when identifying an etiology (Jacobson, 2017). Moreover, recent studies have demonstrated the TB-specific antigen-to-phytohemagglutinin (TBAg/PHA) ratio for T-SPOT. TB (T-SPOT) assay has potential value in discriminating ATBI from LTBI (Wang et al., 2016; Bosco et al., 2017; Zhou et al., 2017). However, it is difficult to apply this extensively, particularly in low-income countries or areas, because it is an expensive and complex assay. Radiographic examinations play an important role in judging the activity of PTB (Skoura et al., 2015), Chest X-ray is the most used image examination, but it has been gradually replaced by chest computed tomography (CT) due to the low detection rate of small lesions and concealed lesions (Long et al., 1998; Skoura et al., 2015). Chest CT had received more and more attention in diagnosing APTB (Bhalla et al., 2015). Unfortunately, chest CT has some limitations due to its inability to distinguish between approximately 20% of active lesions and 11% of inactive lesions.

In recent years, with the development of analytical approaches, the construction of mathematical models based on multiple markers has been increasingly applied in the field of medicine. A nomogram was used for developing a scoring system to identify malignant pleural effusion, and the scoring system exhibited good diagnostic performance (Wang et al., 2020). Similarly, A promising diagnostic model to discriminate between active tuberculosis and latent tuberculosis infection was constructed based on machine learning (Luo et al., 2022). All approaches combine a series of significant parameters to generate a predictive model to achieve better diagnostic performance.

In a variety of previous studies, routine blood examination indices and routine biochemical indicators commonly used in clinical practice have been proven to be of minor or moderate value in the diagnosis of TB (Luo et al., 2020; Luo et al., 2021a; Luo et al., 2021b). However, a single diagnostic indicator is insufficient owing to its poor sensitivity and specificity. This study attempted to construct a predictive model based on routine laboratory parameters to differentiate between APTB and IPTB. The predictive model was shown to be a nomogram, which was then transformed into a scoring system for use in clinical practice and easy clinical application.

## Material and methods

### Study design

**Figure 1:**
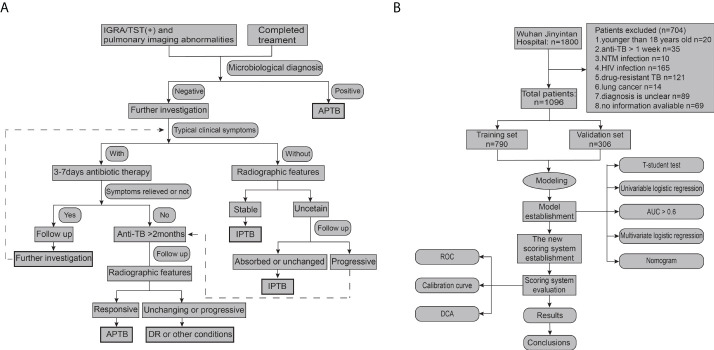
Flowchart of participant selection and the performance of the steps **(A)**; Flowchart of the diagnostic criteria for APTB and IPTB **(B)**. APTB, active pulmonary tuberculosis; IPTB, inactive pulmonary tuberculosis; NTM, non-tuberculous mycobacteria; DCA, decision curve analysis; ROC, receiver operating characteristic. AUC, area under the curve; DR, drug resistance.

This retrospective clinical study was performed between January 2017 and December 2019 at the Wuhan Jinyintan Hospital, Tongji Medical College, Huazhong University of Science and Technology (Infectious Disease Hospital). The clinical features and radiological and laboratory examination results of all patients were collected. All patients were diagnosed with APTB or IPTB by three senior TB specialist doctors. SPSS software (version 20.0; IBM, Chicago, IL, USA) was used to divide all participants into training and validation cohorts according to a ratio of 7:3. 70% of PTB individuals are designated as training cohort, and 30% are validation cohort. A diagnostic model and scoring system to differentiate APTB from IPTB were constructed from those in the training cohort. The diagnostic performance of the scoring system was validated in a validation cohort. The diagnosis of APTB included microbiological and clinical diagnosis according to the Chinese expert consensus. (Deng and Lu, 2020), and the microbiological diagnostic criteria were considered as the positive results of smear microscopy, M. tuberculosis cultures, or nucleic acid amplification (NAA) assays (such as PCR and Xpert MTB/RIF). IPTB is described as abnormal stable radiography results in a person with a positive tuberculin skin test (TST) or interferon-release assay (IGRA), negative bacteriologic assay (if performed), and no clinical and/or radiographic evidence of current disease (after excluding other infections). The detailed diagnostic process for APTB and IPTB is shown in **Figure 1A**. The exclusion criteria were as follows: age < 18 years, indeterminable PTB activity, existing missing value during data collection, more than 1 week of anti-TB treatment, HIV infection, known primary immunodeficiency or immunosuppressive medication, drug-resistant TB, lung cancer, and nontuberculous mycobacterial infection.

### Data collection

Demographic variables (age and sex), objective symptoms (fever, cough, chest pain, night sweats, etc.), and the results from radiographic and laboratory examinations, such as routine blood examinations, biochemical measurements, and microbiological examinations, were all obtained from the electronic medical record (EMR) system. In addition, several ratios, including the monocyte-to-high-density lipoprotein ratio (MHR), lactate dehydrogenase-to-adenylate dehydrogenase ratio (LAR), high-sensitivity C-reactive protein-to-albumin ratio (HSCAR), high-sensitivity C-reactive protein-to-prealbumin ratio (HSCPR), platelet-to-lymphocyte ratio (PLR), neutrophil-to-lymphocyte ratio (NLR), monocyte-to-lymphocyte ratio (MLR), and high-sensitivity C-reactive protein-to-lymphocyte ratio (HSCLR), were calculated and included in the present study.

### Statistical analysis

SPSS software (version 20.0; IBM, Chicago, IL, USA) was used to perform a statistical analysis of the data. Student’s t-test or Mann-Whitney U test was used to compare the continuous variables between groups, the results of which are presented using the mean ± standard deviation (SD) or median (interquartile range, IQR), and the chi-square (2) test was used to compare the categorical variables between groups, the results of which are presented as the absolute value (n) and percentage (%). Additionally, the cutoff values for the reference ranges of the laboratory indices or the optimal cutoff values of the receiver operating characteristic (ROC) curves were used to transform all continuous variables into binary variables. In the training cohort, univariate logistic regression analysis and ROC analyses were performed to identify the variables with a P-value of <0.05 and an area under the curve (AUC) value greater than 0.6. Subsequently, significant variables were analyzed using multivariable logistic regression analysis (MLRA), which was performed to construct the predictive model. The nomogram of the scoring system was created using the RMS tool in R and independent variables in the MLRA. The corresponding score of each parameter is based on the regression coefficient in the results of the multivariate logistic analysis, and the specific methods are as follows: (1) Parameter with the largest coefficient in the logistic regression analysis was defined as 100 points, and the scores of the remaining parameters were obtained through the regression coefficient of equal ratio calculation; (2) Dividing all scores by 10, and when there is a non-integer score, we round up the whole number to get the final score. The model score was automatically computed and evaluated for each patient using the ROC curve analysis. A two-sided p value was computed, and statistical significance was defined as p < 0.05.

## Results

### Patient characteristics

A total of 1096 patients with PTB admitted to Wuhan Jinyintan Hospital were included in this study. Of these patients with PTB, 744 were included in the training cohort (70%; 458 patients with APTB and 286 patients with IPTB) and 352 were included in the validation cohort (30%; 220 patients with APTB and 132 patients with IPTB). A detailed flowchart of the participant selection protocol and steps performed in this study are shown in **Figure 1B**.

There were no significant differences in age and sex among different groups. Additionally, the number of APTB patients with typical symptoms was significantly higher than those of IPTB patients in the training and validation set (P<0.001). Importantly, the number of APTB with Diabetes mellitus (DM) also was significantly higher than those in IPTB patients with DM in the training set (P=0.033), however, there was no significant difference between the number of APTB with DM and IPTB with DM in the validation set (P=0.204). The results suggested that APTB patients were more prone to comorbid DM. The clinical and demographic characteristics of patients are shown in **Table 1**.

**Table 1:** Demographic, clinical, and laboratory characteristics of the training set and validation set.

Variables	training set (744)			validation set (352)			P
	APTB (458)	IPTB (286)	P	APTB (220)	IPTB (132)	P	
**Age (years)**	44.84 ± 18.092	46.56 ± 17.65	0.179	45.88 ± 17.21	47.77 ± 16.78	0.258	0.316
**Sex, Male (%)**	310 (67.7)	177 (61.9)	0.106	140 (63.6)	86 (65.2)	0.774	0.685
**Typical symptoms (%)**	423 (92.4)	129 (45.1)	<0.001	201 (91.4)	71 (53.8)	<0.001	0.318
**Microbiological test, positive (%)**	275 (60.0)	NA	NA	127 (57.7)	NA	NA	0.765
**Underlying condition or illness**							
Diabetes mellitus (%)	50 (10.9)	18 (6.3)	0.033	28 (12.7)	11 (8.3)	0.204	0.312
Positive for HbsAg (%)	38 (8.3)	18 (6.3)	0.314	20 (9.1)	8 (6.1)	0.309	0.804
Other infections (%)	76 (16.6)	46 (16.1)	0.855	47 (21.4)	22 (16.7)	0.283	0.192
Others (%)	155 (33.8)	114 (39.8)	0.097	77 (35.0)	54 (40.9)	0.267	0.734
NA (%)	139 (30.4)	90 (31.5)	0.748	48 (21.8)	37 (28.0)	0.187	0.023

Typical symptoms: cough> 2 weeks, expectoration, fever, hemoptysis, chest pain, weight loss, anhelation and night sweat. Microbiological tests: smear microscopy, M. tuberculosis cultures, and nucleic acid amplification (NAA) assays (such as PCR and Xpert MTB/RIF). Other infections: bacterial, mycotic (diagnosed by microbiological evidence), and mycoplasma infections (positive for mycoplasma IgM). Others: bronchiectasis, hypertension, coronary heart disease, gallbladder polyps, and thyroid nodules.

### Construction of the nomogram and scoring system

**Figure 2:**
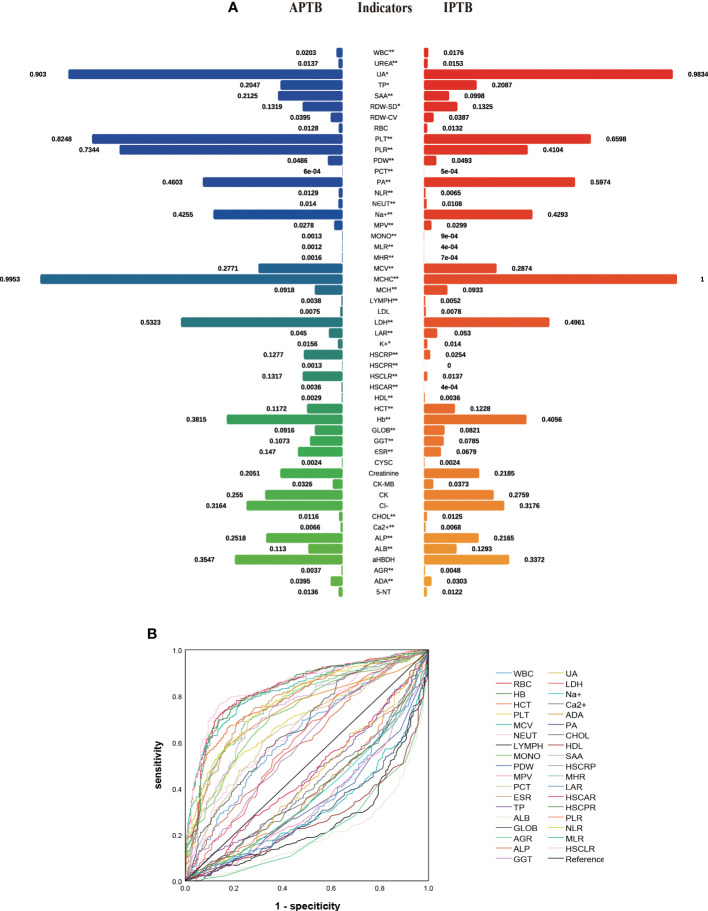
The performance of parameters in the training cohort. **(A)** The comparison between APTB and IPTB in the training cohort. The values represented the median after normalization to range between 0 and 1. *P < 0.05 and **P < 0.001; **(B)** The ROC analysis for significant parameters in univariate logistic regression analyses. Curves in the upper indicated that the levels of these indicators are higher in APTB than in IPTB. Curves in the bottom indicated that the levels of these indicators are lower in APTB than in IPTB.

**Figure 3:**
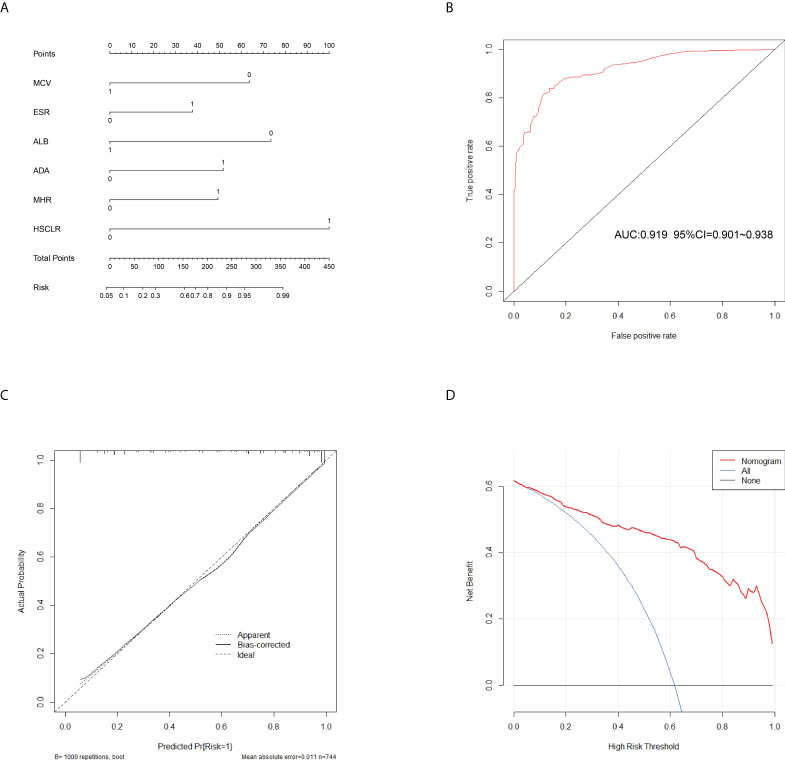
Calibration and clinical use of a diagnostic nomogram for the identification of APTB and IPTB. **(A)** Diagnostic nomogram for discriminating APTB from IPTB. **(B)** The ROC analyses for the Diagnostic model. **(C)** Calibration curve of the diagnostic nomogram. **(D)** DCA of the diagnostic nomogram.

In the training cohort, 41 parameters in this study were significantly different between the APTB and IPTB groups using the Student’s t-test or Mann-Whitney U test (**Figure 2A**). Then, the univariate logistic regression analyses showed that there are 37 parameters that had significant differences (**Supplementary Table 1**). According to the P-value of <0.05 and an area under the curve (AUC) value greater than 0.6, the ROC analyses were further implemented to select significant indices (**Figure 2B**). Finally, the MLRA was conducted to construct the diagnostic model. The exclusion criteria of the model variables are based on the following three conditions: (1) the variable had no significant difference in MLRA; (2) existing collinearity; (3) the variable such as PDW had a significant difference in MLRA, but its exclusion did not affect the diagnostic power of the model. The results demonstrated six parameters mean corpuscular volume (MCV), erythrocyte sedimentation rate (ESR), albumin level (ALB), adenosine deaminase level (ADA), MHR, and HSCLR were important and were included in the MLRA (**Table 2**). We then integrated these six parameters to construct a nomogram to distinguish between APTB and IPTB (**Figure 3A**). The diagnostic nomogram possessed a high discriminative power (AUC = 0.919; 95% CI:0.901-0.938; sensitivity, 0.841; specificity, 0.863) (**Figure 3B**) and good calibration (**Figure 3C**). Furthermore, decision curve analysis (DCA) demonstrated that the nomogram in the present study was advantageous in differentiating between patients with APTB and patients with IPTB, which indicated that patients with PTB using the nomogram demonstrated more clinical benefit than did patients considered as having APTB or IPTB, with a threshold probability of 0.62 (**Figure 3D**).

**Table 2:** Significant indexes in the multivariable logistic regression analyses.

Variables	HR (95%CI)	P Value
MCV	0.27 [0.16, 0.43]	<0.05
ESR	2.19 [1.39, 3.44]	<0.05
ALB	0.22 [0.14, 0.34]	<0.05
ADA	2.93 [1.82, 4.76]	<0.05
MHR	2.79 [1.79, 4.38]	<0.05
HSCLR	8.01 [5.07, 12.85]	<0.05

MCV, mean corpuscular volume; ESR, erythrocyte sedimentation rate; ALB, albumin; ADA, adenylate dehydrogenase; MHR, monocyte-to-high-density lipoprotein ratio; HSCLR, high-sensitivity C-reactive protein-to-lymphocyte ratio.

This nomogram is easy to use in clinical practice in TB high burden countries, especially in remote areas and economically underdeveloped areas. A scoring system was established based on the nomogram with integer points as follows: MCV (6 points), ESR (4 points), ALB (7 points), ADA (5 points), MHR (5 points), and HSCLR (10 points) (**Table 3**).

**Table 3:** A scoring system developed from a nomogram of the training set.

Parameters	Score generated from nomogram/10 (points)	Score modified from nomogram/10 (points)
MCV (<91)	6.36	6
ESR (≥27.2)	3.75	4
ALB (≤40)	7.35	7
ADA (≥12)	5.17	5
MHR (≥0.352)	4.93	5
HSCLR (>5.75)	10	10

MCV, mean corpuscular volume; ESR, erythrocyte sedimentation rate; ALB, albumin; ADA, adenylate dehydrogenase; MHR, monocyte-to-high-density lipoprotein ratio; HSCLR, high-sensitivity C-reactive protein-to-lymphocyte ratio.

### Diagnostic performance of the scoring system in the training cohort

**Figure 4:**
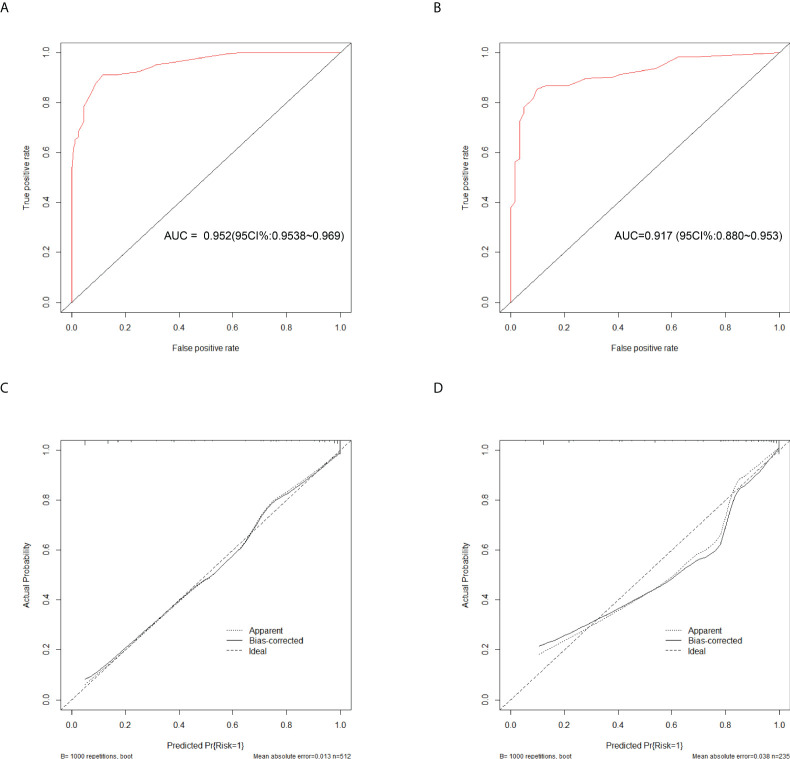
Discrimination and calibration of the scoring system for discrimination of APTB and IPTB. ROC curves of the scoring system in the training cohort **(A)** and validation cohort **(B)**. Calibration curves of the scoring system in the training cohort **(C)** and validation cohort **(D)**.

To evaluate the diagnostic performance of the scoring system, the specific score of each parameter was integrated to obtain the total score. Then the ROC analysis was carried out. The total score corresponding to the maximum Jordan index was selected as the cut-off value. **Table 4** demonstrates that the scoring system had an optimal diagnostic performance when the cutoff value for total points was 15.5, which indicates that the patients with PTB had a higher possibility of APTB when the total point score was >15.5, whereas the patients with PTB had a lower possibility of APTB when the total point score was <15.5 points). The AUC, sensitivity, and specificity were 0.919 (95% CI:0.901-0.938) (**Figure 4A**), 84.06%, and 86.36%, respectively (**Table 4**). Furthermore, this scoring system exhibited satisfactory calibration between the prediction probabilities of the scoring system and the actual probabilities (**Figure 4C**).

**Table 4:** ROC analysis of the scoring system for identifying APTB in the training set.

Cutoff score	Youden index	Sensitivity% (95%CI)	Specificity% (95%CI)	Likelihood ratio
> 13.5	0.684	87.99 (84.66% to 90.82%)	80.42 (75.34% to 84.86%)	4.494
> 14.5	0.689	86.03 (82.51% to 89.07%)	82.87 (77.99% to 87.05%)	5.021
> 15.5	0.705	84.06 (80.38% to 87.29%)	86.36 (81.83% to 90.12%)	6.164
> 16.5	0.702	81.66 (77.81% to 85.1%)	88.46 (84.18% to 91.92%)	7.077
> 17.5	0.664	75.76 (71.57% to 79.62%)	90.56 (86.56% to 93.69%)	8.025

### Diagnostic performance of the scoring system in the validation cohort

This scoring model exhibited good discriminative power in the validation cohort, as evidenced by an AUC of 0.900 (95% CI:0.869-0.931) (**Figure 4B**). When the ideal cut-off point was set at 15.5, the relevant specificity and sensitivity values were 82.73% and 86.36%, respectively (**Supplementary Table 2**). Furthermore, the scoring system demonstrated satisfactory calibration in the validation cohort (**Figure 4D**).

## Discussion

APTB and IPTB represent two PTB states. However, existing laboratory evaluations have limitations, which limit differential diagnosis using conventional methods. Moreover, the clinical treatment and outcomes were dissimilar. Therefore, it is essential to discriminate between these two PTB states. Thus far, research has mainly focused on identifying ATBI and LTBI. Luo (Luo et al., 2020) established a diagnostic model based on the TBAg/PHA ratio and iron metabolism indices for the differential diagnosis of ATBI and LTBI, with a sensitivity and specificity of 88.80% and 91.09%, respectively. Similarly, a predictive model based on routine laboratory indicators to differentiate between ATBI and LTBI, which has high diagnostic ability, exhibited an AUC, sensitivity, and specificity of 0.9880, 92.72%, and 95.99%, respectively (Luo et al., 2022). Although the predictive model has high diagnostic efficiency, it involves 15 indices and is not convenient for clinical practice. Unfortunately, there is no specific method to distinguish between APTB and IPTB. Hence, our study is the first to develop a scoring system based on routine blood examination indices and routine biochemical indicators (MCV, ESR, ALB, ADA, MHR, and HSCLR) to discriminate APTB from IPTB. Moreover, other advantages of the present study were that the indices used in the scoring system were easily obtained, had less time cost than those of conventional laboratory microbiological approaches, and had less cost because the indices involved in the scoring system were routine examinations.

Although there are many differences between APTB and IPTB in terms of clinical characteristics, a single feature has limitations in distinguishing between the two different states of PTB because of its low sensitivity and specificity. Recently, with the growth of clinical data and advanced machine learning methodologies, an increasing number of studies have developed mathematical models based on several markers to improve the diagnostic performance for similar diseases (Wang et al., 2020). In the present study, a scoring system for differentiating APTB from IPTB was developed to enhance the diagnostic possibility of APTB or IPTB more easily for clinicians. Hence, the indices in the scoring system established in this study are easy to access, particularly in economically underdeveloped areas or primary hospitals. In the present study, 52 indices were intergraded, including primary indices and informative ratios commonly used in clinical practice, such as MHR and HSCLR. In recent years, MHR and HSCLR have been newly discovered inflammatory markers. Compared with other inflammatory markers, such as interleukins, MHR and HSCLR are simple, easy to measure, and relatively stable. It is worth noting that previous studies mainly used the C-reactive protein-to-lymphocyte ratio (CLR) as the research focus. The present study considered HSCLR due to the higher sensitivity of high-sensitivity C-reactive protein than that of C-reactive protein. Emerging data suggest that higher MHR and CLR values are associated with various diseases and organ dysfunctions. Recent research has demonstrated that MHR can indicate the prevalence of diabetic retinopathy in patients with type 2 diabetes mellitus (Tang et al., 2021). Higher MHR values represent the highest predictive value for the risk of atherosclerosis (Zhou et al., 2021). In addition, the clinical value of CLR has been researched in previous studies on decompensated cirrhosis, colorectal liver metastases (Taniai et al., 2021), and pneumonia (Cillóniz et al., 2021). Interestingly, there is no literature on MHR and HSCLR in TB patients. Our investigation revealed that MHR and HSCLR had greater diagnostic significance in distinguishing between APTB and IPTB, as evidenced by AUC values of 0.721 and 0.799, respectively.

Previous studies have suggested that MCV is a marker of pulmonary inflammation (Abba, 2009) and that MCV levels are negatively correlated with bacillary load in the lungs (Baruch Baluku et al., 2020). ESR is an inflammatory index, which can reflect the state of TB. The levels of ESR in active TB patients were significantly higher than those in the healthy population. Similarly, ESR levels were significantly higher in the ATB group than in LTBI (Luo et al., 2022). The role of ALB in PTB has attracted increased attention. Hypoalbuminemia is positively associated with the severity of PTB clinical manifestations of PTB (Xu et al., 2021). In addition, ALB levels were significantly lower in the ATB group than in LTBI (Luo et al., 2022). Additionally, a previous study suggested that ADA level has a 98% positive predictive value in high TB prevalence areas (Ding and Zhang, 2018). In addition, ADA activity in ATB patients was significantly higher than that in LTBI patients, and the level of ADA activity was significantly decreased after the completion of anti-TB prophylaxis treatment (Tozkoparan et al., 2007). In addition to the indicators involved in this scoring system, other parameters like hemoglobin, and white blood cell (WBC) have shown a significant difference in our study. These results are consistent with the previous studies. Haemoglobin could be used as a chronic consumption index for active TB (Rathish and Siribaddana, 2018). Additionally, the WBC count in TB patients is significantly higher than in healthy individuals, and the absolute WBC counts decreased significantly during Anti-tuberculosis treatment (Chedid et al., 2020). Neutrophil and lymphocyte are subtypes leukocyte. Previous studies have shown that neutrophil count is an independent predictor of the radiologic severity of PTB at the end of the treatment period (Jones et al., 2021) and lymphocytopenia represented critical inflammatory states (Li et al., 2021).

However, our study had some limitations. First, this study was based on retrospective data, and a selection bias may have existed. Therefore, forward-looking external validation is required. Second, because the participants enrolled in this study were aged>18 years, the performance of the diagnostic model in individuals aged <18 years was unclear. Third, changes in MCV, ALB, and ADA levels after Suspicious were not investigated. Finally, although the scoring system showed relatively excellent performance, the reference range of indicators involved in this model may not be extensive because of confounding factors, such as testing instruments, specimen collection, and testing personnel. Further multicenter validation of this scoring system is required.

In conclusion, MCV, ESR, ALB, ADA, MHR, and HSCLR were significant in differentiating between APTB and IPTB. This scoring system achieved good diagnostic performance and calibration for discriminating APTB from IPTB.

## Data availability statement

The original contributions presented in the study are included in the article/**Supplementary Material**. Further inquiries can be directed to the corresponding authors.

## Ethics statement

The studies involving human participants were reviewed and approved by the ethics committee of Wuhan Jinyintan Hospital, Tongji Medical College of Huazhong University of Science and Technology. Written informed consent for participation was not required for this study in accordance with the national legislation and the institutional requirements.

## Author contributions

FG and XW conceived the study. QY, JY, HL, GL, GW, and JM collected patients’ clinical features and radiological and laboratory examination results. QY, WW, and ST were responsible for statistical analysis. QY, JY, and FG interpreted the data and wrote the manuscript. All authors contributed to the article and approved the submitted version.

## Funding

This work was supported by the National Science and Technology Major Special Foundation of China (2020ZX9201001), Scientific Research Fund of Wuhan in Hubei Province (WX16C33), and Scientific Research Fund of Wuhan in Hubei Province (WX12C02).

## Conflict of interest

The authors declare that the research was conducted in the absence of any commercial or financial relationships that could be construed as a potential conflict of interest.

## Publisher’s note

All claims expressed in this article are solely those of the authors and do not necessarily represent those of their affiliated organizations, or those of the publisher, the editors and the reviewers. Any product that may be evaluated in this article, or claim that may be made by its manufacturer, is not guaranteed or endorsed by the publisher.

## Glossary

**Table:**

WBC	white blood cell
UA	uric acid
TP	total protein
SAA	serum amyloid A
RDW-CV	red blood cell volume distribution width
RDW-SD	standard deviation in red cell distribution width
RBC	red blood cell
PLT	platelet
PLR	platelet to lymphocyte ratio
PDW	platelet distribution width
PCT	platelet hematocrit
PA	prealbumin
NLR	neutrophils to lymphocyte ratio
NEUT	neutrophil count
Na^+^	natrium
MPV	mean platelet volume
MONO	monocyte count
MLR	monocyte to lymphocyte ratio
MHR	monocyte to high density lipoprotein Ratio
MCV	mean red blood cell volume
MCHC	mean corpuscular hemoglobin contentration
MCH	mean corpuscular hemoglobin
LYMPH	lymphocyte count
LDL	low density lipoprotein
LDH	lactate dehydrogenase
LAR	Lactate dehydrogenase to Adenylate dehydrogenase Ratio
K^+^	kalium
HSCRP	high sensitivity C-reactive protein
HSCPR	high sensitivity C-reactive protein to prealbumin ratio
HSCLR	high sensitivity C-reactive protein to lymphocyte ratio
HSCAR	high sensitivity C-reactive protein to albumin ratio
HDL	high density lipoprotein
HCT	hematocrit
Hb	hemoglobin
GLOB	globulin
GGT	gama-glutamyl transpeptidase
ESR	erythrocyte sedimentation rate
CYSC	cystatin C
CK	creatine kinase
CK-MB	CK isoenzyme MB
CL^-^	chlorine
CHOL	serum total cholesterol
Ca^2+^	calcium
ALP	alkaline phosphatase
ALB	albumin
αHBDH	α-hydroxybutyrate dehydrogenase
AGR	albumin to globulin ratio
ADA	adenylate dehydrogenase
5-NT	5'-nucleoticlase.
