# Supplementary material for: A scoring system developed from a nomogram to differentiate active pulmonary tuberculosis from inactive pulmonary tuberculosis
Source: Front Cell Infect Microbiol. 2022 Sep 2;12:947954. doi: 10.3389/fcimb.2022.947954 (PMC9478038; doi:10.3389/fcimb.2022.947954)
Supplement: Supplementary file 1 [file Table_1.docx]

**Supplementary Table 1** the univariable logistic regression analyses in the study

| Variables | OR | 95%CI | | P value |
| --- | --- | --- | --- | --- |
|  |  | lower | upper |  |
| WBC | 1.202 | 1.115 | 1.296 | 0 |
| RBC | 0.724 | 0.563 | 0.931 | 0.012 |
| HB | 0.974 | 0.965 | 0.983 | 0 |
| HCT | 0.935 | 0.907 | 0.963 | 0 |
| PLT | 1.011 | 1.008 | 1.013 | 0 |
| MCV | 0.863 | 0.833 | 0.894 | 0 |
| NEUT | 1.388 | 1.257 | 1.531 | 0 |
| LYMPH | 0.191 | 0.138 | 0.264 | 0 |
| MONO | 103.872 | 37.654 | 286.542 | 0 |
| PDW | 0.195 | 0.127 | 0.3 | 0 |
| MPV | 0.582 | 0.504 | 0.672 | 0 |
| PCT | 17346.754 | 1082.57 | 277958.735 | 0 |
| ESR | 1.044 | 1.036 | 1.052 | 0 |
| TP | 0.972 | 0.951 | 0.994 | 0.011 |
| ALB | 0.767 | 0.733 | 0.802 | 0 |
| GLOB | 1.12 | 1.085 | 1.156 | 0 |
| AGR | 0.036 | 0.02 | 0.065 | 0 |
| ALP | 1.011 | 1.006 | 1.017 | 0 |
| GGT | 1.009 | 1.003 | 1.014 | 0.003 |
| UA | 0.998 | 0.996 | 0.999 | 0.002 |
| LDH | 1.007 | 1.003 | 1.011 | 0 |
| Na+ | 0.885 | 0.841 | 0.931 | 0 |
| Ca2+ | 0.121 | 0.043 | 0.339 | 0 |
| ADA | 1.241 | 1.178 | 1.307 | 0 |
| PA | 0.988 | 0.985 | 0.991 | 0 |
| CHOL | 0.694 | 0.586 | 0.823 | 0 |
| HDL | 0.059 | 0.033 | 0.105 | 0 |
| SAA | 1.008 | 1.005 | 1.011 | 0 |
| HSCRP | 1.052 | 1.041 | 1.063 | 0 |
| MHR | 144.336 | 55.031 | 378.562 | 0 |
| LAR | 0.913 | 0.886 | 0.94 | 0 |
| HSCAR | 8.566 | 5.452 | 13.458 | 0 |
| HSCPR | 144.769 | 39.88 | 525.531 | 0 |
| PLR | 1.017 | 1.014 | 1.02 | 0 |
| NLR | 2.151 | 1.85 | 2.501 | 0 |
| MLR | 18148.439 | 3329.469 | 98924.445 | 0 |
| HSCLR | 1.093 | 1.074 | 1.113 | 0 |

**Abbreviations:** WBC: white blood cell; RBC: red blood cell; HB: hemoglobin; HCT: Hematocrit; PLT: platelet; MCV: Mean red blood cell volume; NEUT: Neutrophils; LYMPH:lymphocyte; MONO: monocyte; PDW: Platelet distribution width; MPV: Mean platelet volume; PCT: Platelet hematocrit; ESR: erythrocyte sedimentation rate; TP:Total protein; ALB: albumin ; GLOB: globulin; AGR: albumin to globulin ratio

ALP : alkaline phosphatase; GGT：gama- glutamyl transpeptidase; UA：uric acid; LDH：lactate dehydrogenase; ADA： Adenylate dehydrogenase; PA：Prealbumin; CHOL：Serum total cholesterol; HDL：high density lipoprotein; SAA：Serum amyloid A; HSCRP：High sensitivity C-reactive protein; MHR：Monocyte to High density lipoprotein Ratio; LAR：Lactate dehydrogenase to Adenylate dehydrogenase Ratio; HSCAR：High sensitivity C-reactive protein to Albumin Ratio; HSCPR: High sensitivity C-reactive protein to Prealbumin Ratio; PLR: Platelet to Lymphocyte Ratio; NLR: Neutrophils to Lymphocyte Ratio; MLR: Monocyte to Lymphocyte Ratio; HSCLR：High sensitivity C-reactive protein to Lymphocyte Ratio

**Supplementary table 2** Diagnostic performance of the scoring system in differentiating APTB from IPTB and negative-etiological PTB from IPTB in the training and internal validation sets

| Variable | APTB/IPTB | |
| --- | --- | --- |
|  | Training set | Internal validation set |
| AUC(95%CI) | 0.919(0.901-0.938) | 0.900(0.869-0.931) |
| Sensitivity(95%CI) | 84.06% (80.38%-87.29%) | 82.73% (77.07% -87.48%) |
| Specificity(95%CI) | 86.36% (81.83%-90.12%) | 86.36% (79.31%-91.71%) |

**Abbreviations:**AUC=area under curve; CI=confidence interval.
